# A1M/α_1_-Microglobulin Protects from Heme-Induced Placental and Renal Damage in a Pregnant Sheep Model of Preeclampsia

**DOI:** 10.1371/journal.pone.0086353

**Published:** 2014-01-28

**Authors:** Lena Wester-Rosenlöf, Vera Casslén, Josefin Axelsson, Anneli Edström-Hägerwall, Magnus Gram, Madlene Holmqvist, Martin E. Johansson, Iréne Larsson, David Ley, Karel Marsal, Matthias Mörgelin, Bengt Rippe, Sigurbjörg Rutardottir, Behnaz Shohani, Bo Åkerström, Stefan R. Hansson

**Affiliations:** 1 Department of Infection Medicine, Lund University, Lund, Sweden; 2 Department of Obstetrics and Gynecology, Lund University, Lund, Sweden; 3 Department of Nephrology, Lund University, Lund, Sweden; 4 Department of Laboratory Medicine, Lund University, Malmö, Sweden; 5 Department of Pediatrics, Lund University, Lund, Sweden; UCL Institute of Child Health, United Kingdom

## Abstract

Preeclampsia (PE) is a serious pregnancy complication that manifests as hypertension and proteinuria after the 20^th^ gestation week. Previously, fetal hemoglobin (HbF) has been identified as a plausible causative factor. Cell-free Hb and its degradation products are known to cause oxidative stress and tissue damage, typical of the PE placenta. A1M (α_1_-microglobulin) is an endogenous scavenger of radicals and heme. Here, the usefulness of A1M as a treatment for PE is investigated in the pregnant ewe PE model, in which starvation induces PE symptoms via hemolysis. Eleven ewes, in late pregnancy, were starved for 36 hours and then treated with A1M (n = 5) or placebo (n = 6) injections. After injections, the ewes were re-fed and observed for additional 72 hours. They were monitored for blood pressure, proteinuria, blood cell distribution and clinical and inflammation markers in plasma. Before termination, the utero-placental circulation was analyzed with Doppler velocimetry and the kidney glomerular function was analyzed by Ficoll sieving. At termination, blood, kidney and placenta samples were collected and analyzed for changes in gene expression and tissue structure. The starvation resulted in increased amounts of the hemolysis marker bilirubin in the blood, structural damages to the placenta and kidneys and an increased glomerular sieving coefficient indicating a defect filtration barrier. Treatment with A1M ameliorated these changes without signs of side-effects. In conclusion, A1M displayed positive therapeutic effects in the ewe starvation PE model, and was well tolerated. Therefore, we suggest A1M as a plausible treatment for PE in humans.

## Introduction

Preeclampsia (PE) affects up to 3–8% of pregnancies, causing a significant number of maternal and fetal deaths worldwide. Despite intensive research, PE still lacks a safe and effective therapy, as well as a reliable early diagnosis [1].

The general view today is that the disease evolves in two stages. The first stage is characterized by a defective formation of the placenta with reduced utero-placental blood flow as a consequence. This leads to oxidative stress that further aggravates the placental vascular dysfunction [2], [3] and gives rise to vascular inflammation and insufficient blood perfusion of the placenta and maternal organs [4], [5].

The second stage of PE is characterized by the clinical manifestations, hypertension and proteinuria, appearing from 20 weeks of gestation and onwards. As the disease progresses, angiospasm in the brain and brain edema may cause severe epileptic seizures–eclampsia [6]. The only cure is delivery of the fetus and removal of the placenta. As placenta removal is crucial for symptom resolution, a placental derived factor has been suggested as a culprit [1].

Increased expression and leakage of one or more placental factors may be the missing link between stage one and two in PE [7]. Our previous results, based on gene and protein profiling, have revealed an increased production and accumulation of cell-free fetal hemoglobin (free HbF) in the PE placenta [8]. Free Hb is well known to have pro-inflammatory, pro-oxidative, tissue damaging and vasoconstrictive properties [9], [10]. Our recent results show that free Hb causes damage to the placental barrier and leads to leakage of free HbF into the maternal blood circulation [11], [12]. Elevation of free HbF is seen as early as at 14 weeks of gestation, thus 2 months or more before onset of the clinical symptoms in those women who will develop PE [13].

Free HbF is autoxidized to metHb and superoxide then further metabolized into free iron and heme. These molecules spontaneously form reactive oxygen species (ROS) and cause oxidative stress, vasoconstriction, kidney damage, endothelial damage and further hemolysis [9]. This constellation of problems is seen in PE and in its more acute form, the HELLP syndrome (**H**emolysis, **E**levated **L**iver enzymes and **L**ow **P**latelets) [2], [14], [15].

Several endogenous defense systems have evolved to protect from the harmful effects of free Hb. The plasma proteins haptoglobin, hemopexin [16], [17] and the plasma- and tissue protein A1M (α_1_-microglobulin) [18]–[20], cooperate to minimize free Hb-induced tissue damages *in vivo*. A1M belongs to the Lipocalin protein family, a group of proteins with a highly conserved three-dimensional β-barrel structure found in animals, plants and prokaryotes [21]. The protein is mainly synthesized in the liver, released into the bloodstream, and then rapidly distributed to the extra-vascular compartment of all tissues [22]. A1M is a reductase and a scavenger of free heme and radicals that protects cells and extracellular matrix against Hb-, heme- and oxidative stress-induced damage [18]–[20]. The expression of A1M is up-regulated by elevated levels of free Hb, heme and ROS [23]. In our previous studies, we have reported increased levels of A1M in plasma and urine of women with PE [12], [13].

We hypothesise that the endogenous production of A1M falls short to meet the increased oxidative stress and the free HbF levels seen in PE. By supplementing with recombinant A1M intravenously, this shortage can be relieved. To evaluate the therapeutic effects of A1M *in-vivo*, a ewe PE model in which increased levels of circulating free Hb causes the typical symptoms was chosen [24], [25]. Starvation of pregnant ewes, causes hemolysis leading to increased levels of free heme in the circulation and development of PE-like symptoms, i.e., hypertension and renal disturbances with proteinuria thus suggested as a good PE model [26]. Further support for the role of heme in the disease mechanism of this model is that the observed symptoms can be ameliorated by decreasing the hemolysis [27]. In this study A1M was evaluated as a potential therapeutic drug for PE by decreasing the effects of free Hb.

## Materials and Methods

### A1M

Recombinant human A1M (A1M) was prepared by A1M Pharma AB (Lund, Sweden) and was dissolved in 20 mM Tris-HCl, pH 8.0, 0.25M NaCl at a concentration of 1.5 mg/mL. A1M contains the full polypeptide of plasma A1M proceeded by an N-terminal His_8_-tag [28]. It was tested and found to be fully functional (reduction of the ABTS-radical, described in [29] and the endotoxin content was <5 EU/mg. The A1M solution was sterile filtered and frozen at −20°C until injection into the ewes. For the placebo animals, a sham solution containing endotoxin-free, sterile 20 mM Tris-HCl, pH 8.0, 0.25M NaCl was used.

### The ewe model

The study was approved by the ethical committee for animal studies at Lund University, permission no: M287-11. A total of 15 ewes, 2–8 years old, from the fifth generation intercross of meat breeds and Dorset, were date-mated and pregnancy was confirmed by ultrasound. Gestational age was calculated from the day of conception. Pregnant ewes at a gestational age of 125–130 days were transferred to the animal facility of Lund University, and randomized concerning gestation day, age and weight of the ewes to the study groups. Personnel performing the study, data collection and analyses were blinded to treatment.

**Figure 1:**
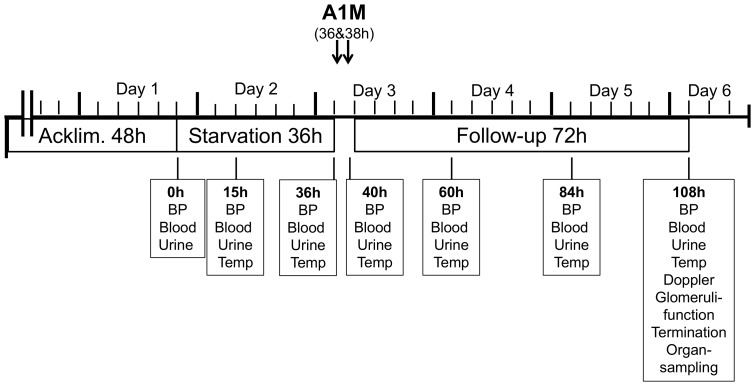
Overview of the experimental setup. The experiment extended over 6 days (108 hours), and the first time-point (0 h) represents the start of starvation. Pre-experimental acclimatization, starvation and follow-up were done at the time-periods shown. A1M-infusion was done twice with a 2-hour interval just before starvation end at 36 hours and 2 hours later as described in the materials and methods section. Sampling was performed at 7 time-points: 0 h, 15 h, 36 h (during starvation) and at, 40 h, 60 h, 84 h and 108 h (corresponding to 4, 24, 48 and 72 hours of the re-fed follow-up period).

All 15 ewes were acclimatized for 48 hours (Figure 1). During this time they were fed daily with silage and water *ad libitum*. Following acclimatization, PE-like symptoms were induced in 11 ewes by a 36 hours starvation period. During the same 36 hours the remaining 4 ewes were fed according to their habits and used as healthy reference controls. At the end of the starvation period, 5 ewes from the starved group were injected intravenously with two bolus doses of A1M. Each dose was administered intravenously in a total volume of 50 mL buffer solution containing 1.8 mg A1M/kg body weight. The second dose was administered 2 hours after the first one. Following the same administration schedule, the remaining 6 ewes in the starvation group were injected intravenously with two 50 mL doses of buffer solution. The 4 control ewes, not exposed to starvation, were sham injected with buffer only (n = 2) or with A1M (n = 2) according to the same protocol as the starved animals. After injections, food was re-introduced to the starved ewes, and all animals were then fed and observed for an additional 72 hours. The ewes were sacrificed at 108 hours (on day 6). Throughout the 108 hours, the animals were monitored for blood pressure, body temperature and for various blood and urine parameters at time-points shown in Figure 1. At 108 hours, Doppler velocimetry of the maternal uterine artery and the umbilical artery was performed, the glomerular filtration rate and sieving coefficients for Ficoll_10–80Å_ were evaluated, and finally after termination, whole blood, kidney and placenta tissue samples were collected.

### Blood and urine analysis

Throughout the 108 hours of the experiment (36 hours of starvation + 72 hours of follow-up), blood pressure (BP) was measured non-invasively using an oscillometric monitor and a tail cuff.

The venous blood and urine samples were collected at 0, 15, 36 of starvation and 4, 24, 48 and 72 hours of follow up. The 36 h time point was just before infusion of A1M/buffer solution and re-feeding the 4 h follow-up time point was approximately two hours after the last bolus dose (Figure 1). An additional blood sample was taken immediately before the second injection (2 hours after the first) in order to follow the kinetics of A1M. The body temperature was measured rectally before and after the injections and thereafter daily until termination. The BP measurements were performed on ewes standing in a box in a calm environment. A cuff was placed at the base of the tail and the systolic and diastolic blood pressure was recorded during 30 minutes using an oscillometric monitor (Lifewindow 6000 V, Digicare Biomedical technology Inc, FL, USA). Heart rate and mean arterial pressure (MAP) were calculated by the algorithm of the monitor. Urine samples were analyzed fresh with Combur^_7^Test sticks read in Urisys 1100 (Roche) and then frozen at −80°C for subsequent analyses. Venous blood was collected in serum-, EDTA- and Li-Heparin tubes and immediately analyzed for glucose and blood cell count. Serum and plasma were separated. The clinically used biochemical PE markers creatinine and liver transaminases in plasma were analyzed and the remaining samples stored at -80°C for subsequent analyses.

### Evaluation of the uteroplacental blood flow with Doppler velocitometry

At 108 hours, Doppler velocimetry measurements were obtained from the maternal uterine and umbilical artery using a Philips IU22 ultrasound system (Philips, Bothells, WA) with a linear array trans-abdominal transducer L12-5. During the procedure, awake, non-sedated ewes were standing in a cage in a dark examination room.

A distal, major ascending branch of the left side uterine artery was localized with color Doppler and a sample volume size chosen to cover the entire vessel cross-section (usually 1.5 mm). The insonation angle was kept below 45° and the wall filter below 120 Hz. Pulsed wave Doppler ultrasound with 6 MHz frequency was used to record the Doppler spectrum of at least 5 heart beats with a uniform waveform appearance. The resistance index (RI according Pourcelot) was calculated automatically by the system in the frozen image [30]. The presence or absence of early diastolic notch, indicating an increased resistance to flow in the uteroplacental circulation, was evaluated qualitatively by inspection of the waveforms. The spectral Doppler examination was repeated 2–4 times.

Subsequently, the free portion of the umbilical cord was localized with color Doppler within the uterine cavity and spectral Doppler signals of umbilical artery blood velocities were recorded and analyzed as for the uterine artery. In the case of twin fetuses, the umbilical artery of the fetus located in the left part of the uterus was examined.

Doppler examinations were performed in 4 of the A1M and in 6 of the placebo treated ewes. In one case from the A1M group, no acceptable and reproducible Doppler signals from the uterine artery were obtained. The results were statistically evaluated using Mann-Whitney rank test or Fisher exact test. Possible associations between the uterine artery RI and maternal mean blood pressure and heart rate, respectively, and the umbilical artery RI and fetal heart rate were tested using Pearson's coefficient of correlation.

### Surgery, termination of animals and samples collected during surgery

Pregnant ewes were intubated following intravenous Thiopentone induction of anesthesia and laparotomy was performed under intravenous analgesia and sedation with repeated doses of Fentanyl and Propofol. Laparotomy was performed with the ewe in dorsal position, the liver and the left kidney were resected and a venous blood sample was collected by puncture of the jugular vein. Following hysterectomy, a fetal arterial blood sample was collected from the umbilical artery. Blood samples from the ewe and lambs were collected in serum-, EDTA-, Li-Heparin and PAX-tubes. Lambs were delivered and placental cotyledons were resected. Following delivery, the gender, weight and number of lambs were registered. Lambs were euthanized by an intracardiac injection of Thiopentone followed by potassium chloride. Obtained tissues from the ewe and lambs respectively were immediately dissected and fresh-frozen, paraffin-embedded and fixed for electron microscopy as appropriate. The ewe was euthanized by an intravenous over-dose of Thiopentone.

### Acute analyses

The blood samples were analyzed for glucose using contour sticks and device (Bayer Consumer Care AG, Basel, Switzerland). The urine samples were tested for pH and ketones using Combur^_7^Test sticks read in an Urisys 1100 (Roche Diagnostics GmbH, Germany). The urine was also tested for protein, leukocytes and erythrocytes/hemoglobin content immediately after sampling with Combur^_7^Test sticks read in Urisys 1100 (Roche).

### Kidney function analyses

Immediately after anesthesia and intubation, FITC-Ficoll was injected intravenously as a single dose. A mixture of FITC-Ficoll 70 (50 mg/mL) and FITC-Ficoll 400 (50 mg/mL) (TdB Consultancy, Uppsala, Sweden), 1∶24, together with FITC-Inulin (10 mg/mL) (TdB Consultancy, Uppsala, Sweden) was used. The single dose contained 3.5 mg of FITC-Ficoll 70, 84 mg of FITC-Ficoll 400 and 62.5 mg of FITC-Inulin in a total volume of 8 mL. Blood sampling in order to evaluate Ficoll content in plasma was performed approximately 22–23 min after the intravenous FITC-Ficoll injection. Urine was sampled at 25 min after Ficoll-injection by urinary bladder puncture in order to avoid blood contamination. FITC-Ficoll Glomerular Sieving Coefficients were determined as previously described [31], [32]. Statistical difference was tested using t-test for unpaired observations. Plasma creatinine was analyzed on a Cobas instrument by an enzymatic colorimetric method.

### Hemolysis and oxidation marker analyses

The heme concentration was determined in plasma using the QuantiChromTM Heme Assay Kit (GENTAUR BVBA – BIOXYS, Belgium). Plasma bilirubin and Ca^2+^ were analyzed on a Cobas instrument by the principle of Diazonium-conjugation and o-Cresolphtalein complex formation, respectively. Free thiol and sulfide in plasma were analyzed using the Thiol and sulfide quantitation kit (Molecular Probes Inc, USA).

### A1M analysis

The specific content of human A1M in the plasma and urine samples was determined by a radioimmunoassay previously described [33]. The antibody did not cross-react with sheep A1M.

### General blood biomarker analyses

Liver transaminase enzyme activity (ALAT, ASAT) and P-LDH were analyzed on a Cobas instrument. Whole blood collected to EDTA tubes was used to asses blood cell count on a VetScan HM5 instrument set for sheep (Abaxis Inc.,USA).

### Plasma interleukin 6 and TNFα analyses

Plasma interleukin 6 (IL-6) was analyzed using a sandwich ELISA with antibodies from AbD Serotec, UK. Monoclonal antibody MCA1659 was used for coating (5 µg/mL) and polyclonal antibody AHP424 for detection of IL-6 (dilution 1∶500) followed by HRP-conjugated swine anti-rabbit IgG (DAKO A/S, Denmark). The procedure followed mainly the description in [34]. The plasma samples were diluted 2-fold. The plates were developed with tetramethylbenzidine (Sigma) and reaction stopped with 2M H_2_SO_4_. The optical density of the plates was read on a Wallac 1420 Multilabel Counter. Levels of TNFa were analyzed using a sheep Tumor Necrosis Factor a (TNF-α) ELISA kit (EMELCA Bioscience, the Netherlands). The ELISA was run in duplicates according to the manufacturer's instructions using plasma samples diluted 2-fold in assay buffer.

### Gene expression analysis

Samples of placenta and kidney tissues were collected immediately after euthanizing the animals. 20×10×5-mm cross sections of placenta cotyledons or whole kidneys were snap frozen on dry ice, and stored at −80°C until RNA extraction. Samples of 10 mL venous blood were collected in PAXgene blood RNA tubes (PreAnalytix GmbH, Hombrehtikon, CH, UK) and processed according to manufacturer's instructions. Total RNA was prepared with Trizol-extraction (Invitrogen, Carlsbad, CA, USA) followed by the EZNA Total RNA Kit (OMEGA Bio-Tec, Doraville, GA, USA). Concentration and purity was determined with Nanodrop ND-1000 (Nanodrop Technologies, Wilmington, DE, USA) and integrity was determined by agarose gel electrophoresis. RNA was converted into cDNA by reverse transcription using TaqMan Reverse Transcription Reagents (Applied Biosystems, Foster City, CA, USA) as described [8]. The final concentration of cDNA was10 ng/µL.

Gene transcripts were quantified using qPCR on a StepOnePlus™ Realtime PCR instrument (Applied Biosystems). Gene Expression Primers and probes for 14 selected genes (AMBP, CAT, HbA, HbF, HGF, HMOX1, IL-6, IL-10, PLGF, sFLT1, SOD1, SOD2, TGFβ, VEGFA) were ordered using the Custom Design service of Applied Biosystems (primer and probe sequences according to Table S1 in file S1). The mRNA of most genes had been sequenced in sheep, but the ovine genome is poorly sequenced: Therefore, information from corresponding genes in the highly homologous bovine genome was used to assure that the Ovine TaqMan probes span exon boundaries. The probes were labeled with fluorogenic dye, 6-carboxyfluorescein (FAM). For two genes, A1M and HMOX1, the ovine mRNA has not been sequenced and therefore bovine gene expression assays were used (Applied Biosystems). The PCR reactions were assayed in duplicates as described in [8]. All gene expression assays were tested for amplification of genomic DNA. A calibration curve, obtained by serial 4-fold dilution of the template DNA (80–0.08 ng), was used for quantification. The quantitative value of each sample was normalized to the corresponding value of β-actin and results are presented as relative values. The Mann-Whitney test was used to evaluate the significance of differences between groups. The test was two-sided and 5% level of significance was used.

### Histology

Tissues were fixed in 4% paraformaldehyde, according to routine protocols. Following paraffin embedding, tissues were sectioned at 3 µm and subsequently stained with hematoxylin and eosin. In assessing the renal tissue compartments, besides a general evaluation, signs of endotheliosis and signs of tubular cellular stress and/or necrosis were specifically searched for. Endotheliosis was defined as signs of edematous swelling of the endothelial cells of the renal vasculature. Tubular cellular stress/necrosis was defined as apical blebbing of cell membranes, non-isometric vacuolization of the cytoplasm and cell sloughing into the tubular lumen.

### Transmission electron microscopy

Organ samples, 2×2×2 mm, were fixed for 1 h at room temperature, and then overnight at 4°C in 2.5% glutaraldehyde in cacodylate buffer, pH 7.4. Then, samples were prepared for ultrathin sectioning and transmission electron microscopy as described in [35]. Analyses were carried out by an independent investigator, blinded for the study groups. For quantitative evaluation of tissue damage, the surface areas of mitochondria and extracellular matrix space as well as the ratio of damaged and intact plasma and nuclear membrane stretches were determined for 60 cell profiles. The values for the surface area for these structures were determined using Adobe Photoshop CS6.

## Results

### The ewe model

**Figure 2:**
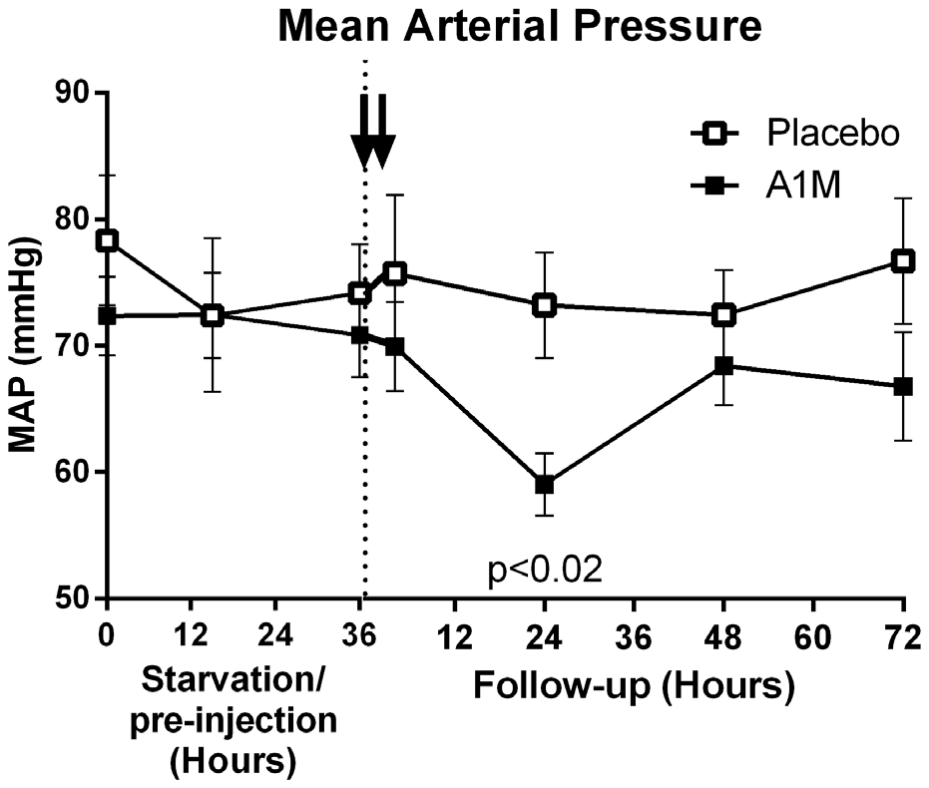
Physiological parameters. The mean arterial blood pressure was monitored non-invasively. The arrows indicate the A1M injections. The data are presented as mean ± SEM. The indicated p-value is valid for the 24 hours re-fed follow-up time point and calculated using un-paired t-test.

The time-flow of the experimental setup, starvation, A1M-infusion and sampling is illustrated in Figure 1. 0 h is defined as the time point just before experimental start. The experiment started with 36 hours of starvation which was followed by A1M or placebo-injections and then follow up for additionally 72 hours. There was no significant difference between the starved placebo-treated or starved A1M-treated groups at the onset of the experiment in terms of age, weight or gestational age (Table S2 in file S1). The frequency of multiple fetuses was slightly higher in the A1M treatment group (Table S3 in file S1). However, no difference in weight per lamb at termination could be observed between the two groups. The starvation caused a statistically significant reduction in blood glucose, urine-pH and plasma Ca^2+^, and an increase in urine ketones at 36 h (Table S4 in file S1). Starvation also led to hemolysis, reflected in a significant increase in plasma bilirubin peaking at 36 h, and a decreased level of free thiol groups in plasma (Table S4 in file S1). The free thiol groups returned to normal levels directly after re-feeding, while bilirubin and calcium returned to normal levels at 48 hours after re-feeding. The blood glucose remained low throughout the experimental period. Starvation did not cause any significant increase in blood pressure or heart rate (Figure 2).

### Effect of A1M treatment

**Figure 3:**
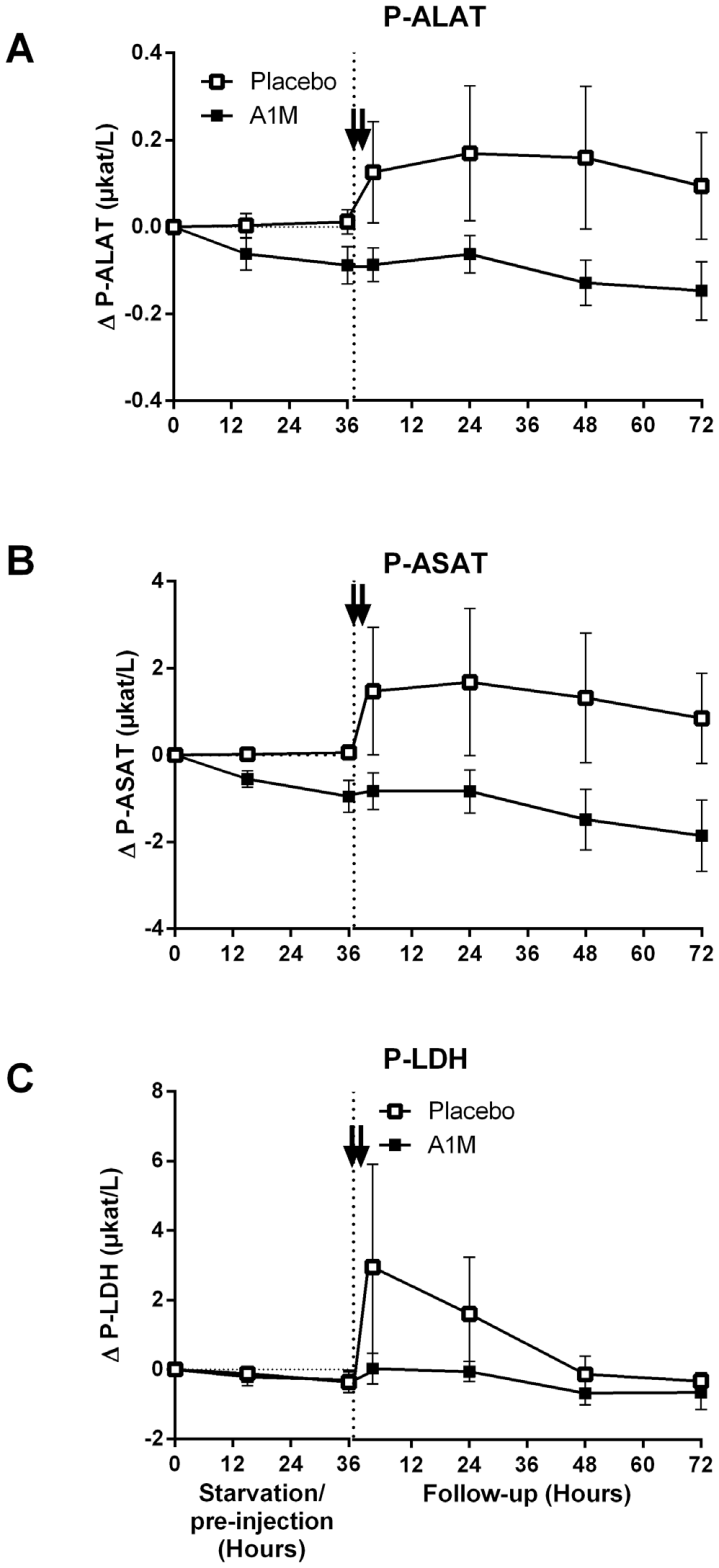
Organ specific markers. ALAT, ASAT and LDH were measured in plasma. The data are presented as mean ± SEM. The arrows indicate the A1M injections. Plasma ALAT, ASAT and LDH showed a strong tendency for up-regulation in the placebo-treated animals compared to A1M treated animals, but no statistical significant changes were obtained. A1M had no effect on any of these markers.

A1M treatment did not change the hemolysis or the number of free thiol groups. However, A1M treatment led to a significant reduction in systolic, diastolic and mean arterial blood pressure 24 hours after the first injection (Figure 2, and Figure S1), but no change in heart rate (Figure S1). The body temperature increased marginally immediately after injections, but no differences were seen between A1M and placebo treatment. For all animals the body temperatures had returned to normal levels of 39.7°C 24 hours after the first injection) (Figure S1). A1M injection did not cause any toxic effects on the liver as measured by liver transaminase (ALAT, ASAT) and LDH levels, which were stable or decreased. There was an increase in ALAT, ASAT and LDH seen after re-feeding in the placebo-treated starved animals, which was not seen in the A1M treated group (Figure 3). However, the difference was not statistically significant.

### A1M pharmacokinetics

**Figure 4:**
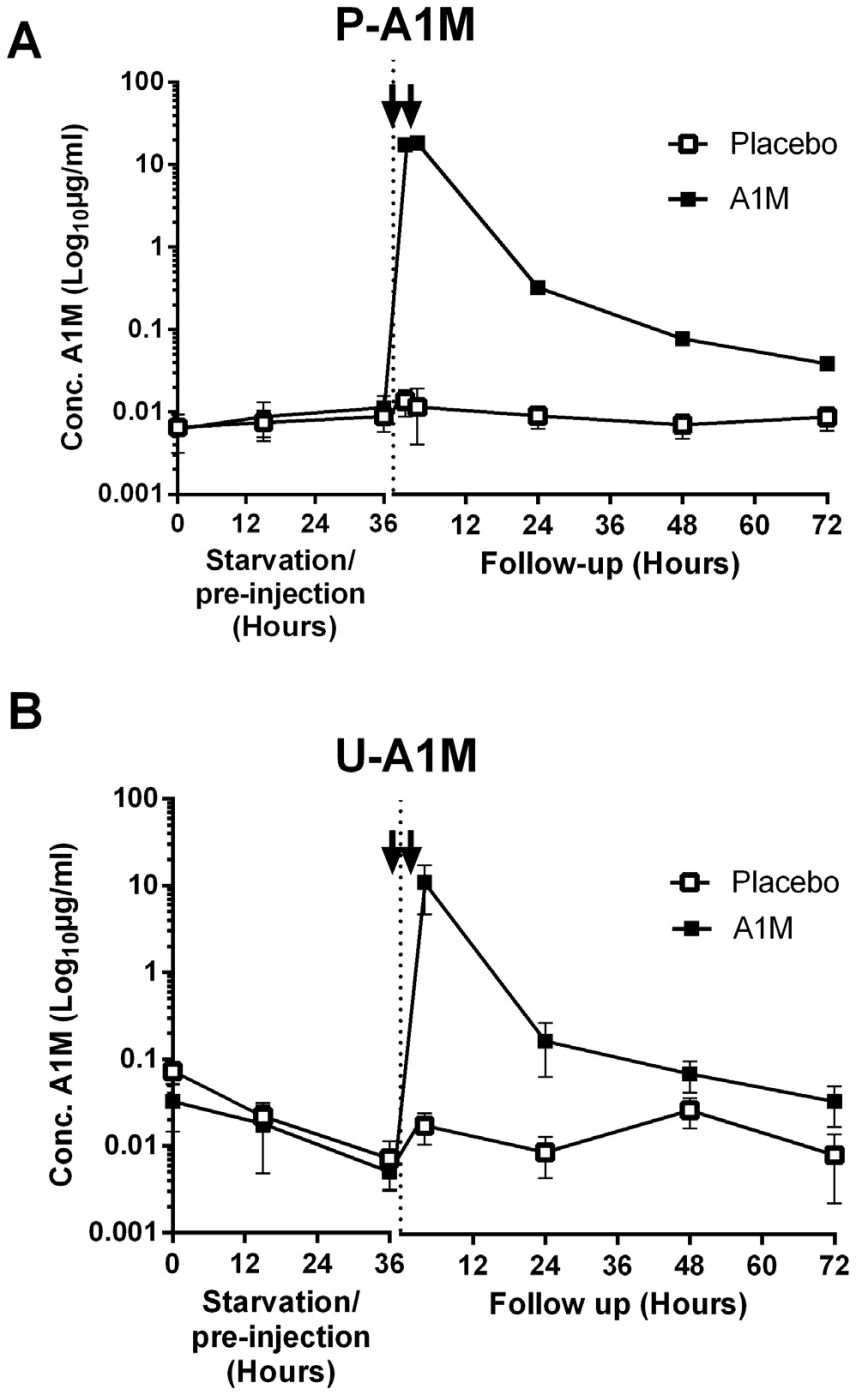
A1M concentrations in plasma and urine. The concentration of injected A1M in plasma (A) and urine (B) was determined by radio-immunoassay specific for human A1M. The concentrations are presented as mean ± SEM on a logarithmic scale.

The injected A1M could be measured specifically without interference from endogenous sheep A1M. The injected A1M showed a quick turn-over both in plasma and urine (Figure 4). In plasma, two hours after the first injection, immediately before the second injection, a concentration of approximately 10 µg/mL A1M was observed corresponding to approximately 18% of the injected first bolus dose. Two hours after the second injection, approximately 11 µg/mL A1M was observed corresponding to 9% of the total injected amount. Accordingly, there was no accumulation of A1M in plasma. 24 hours after the first injection approximately 0.25% of the total injected amount remained in the blood. In urine, injected A1M was seen approximately two hours after the second injection and 24 hours later only 1% of the peak concentration could be detected.

### Placenta histology

**Figure 5:**
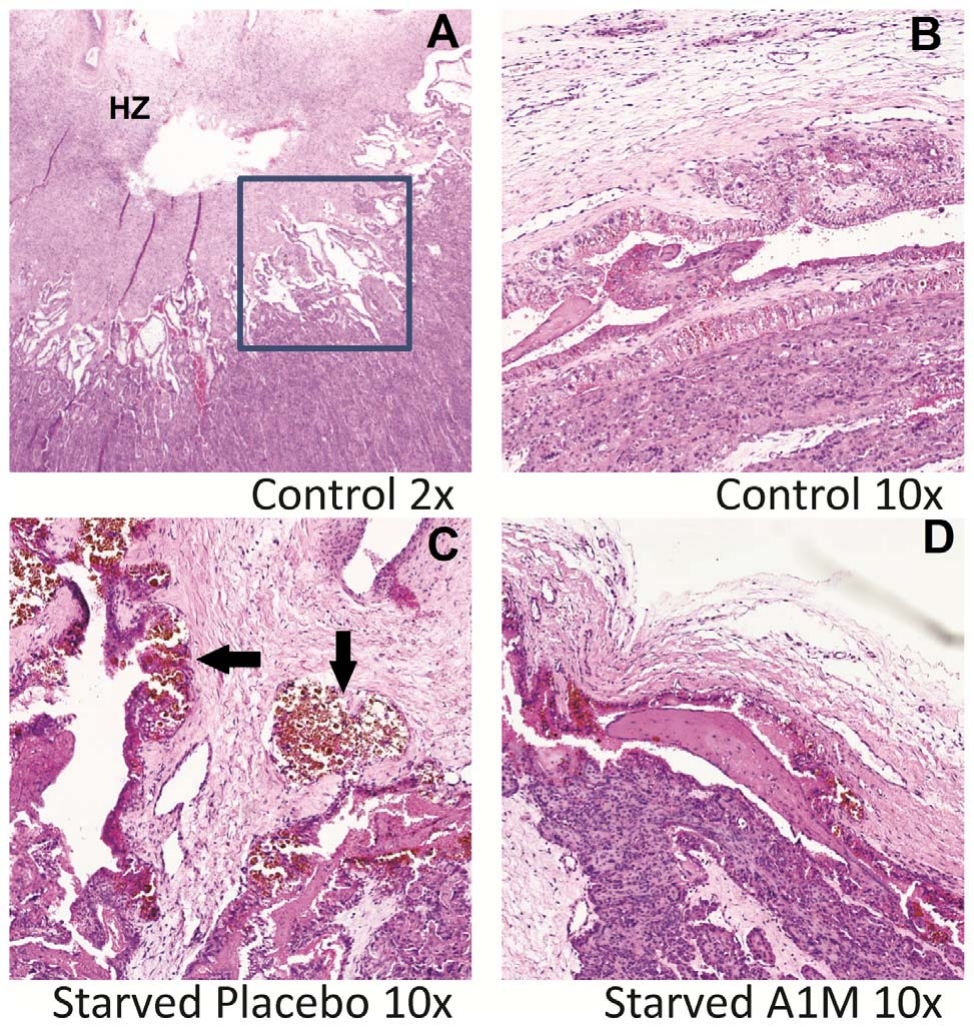
Placenta histology visualized by light microscopy. The histology of placenta was assessed in light microscopy of HE-stained slides. A. HE-stained placentome of a healthy control ewe at gestation d135 (2× magnification). The hilar zone is marked (HZ) and the square show the haemophagus area where differences were observed. B. HE stained haemophagus area of the placentome from a representative control ewe (10× magnification). C. HE stained haemophagus area of the placentome from a representative starved placebo ewe (10× magnification). Areas with increased erythrophagocytosis are marked with arrows. D. HE stained haemophagus area of the placentome from a representative starved A1M-treated ewe. Note the decreased amount of erythrocyte debris.

The placenta histology revealed an increased erythrophagocytosis in the starved placebo treated animals compared to controls. A1M-treatment appeared to attenuate phagocytosis (Figure 5). The remaining parts of the placentome was unaffected by starvation and treatment.

**Figure 6:**
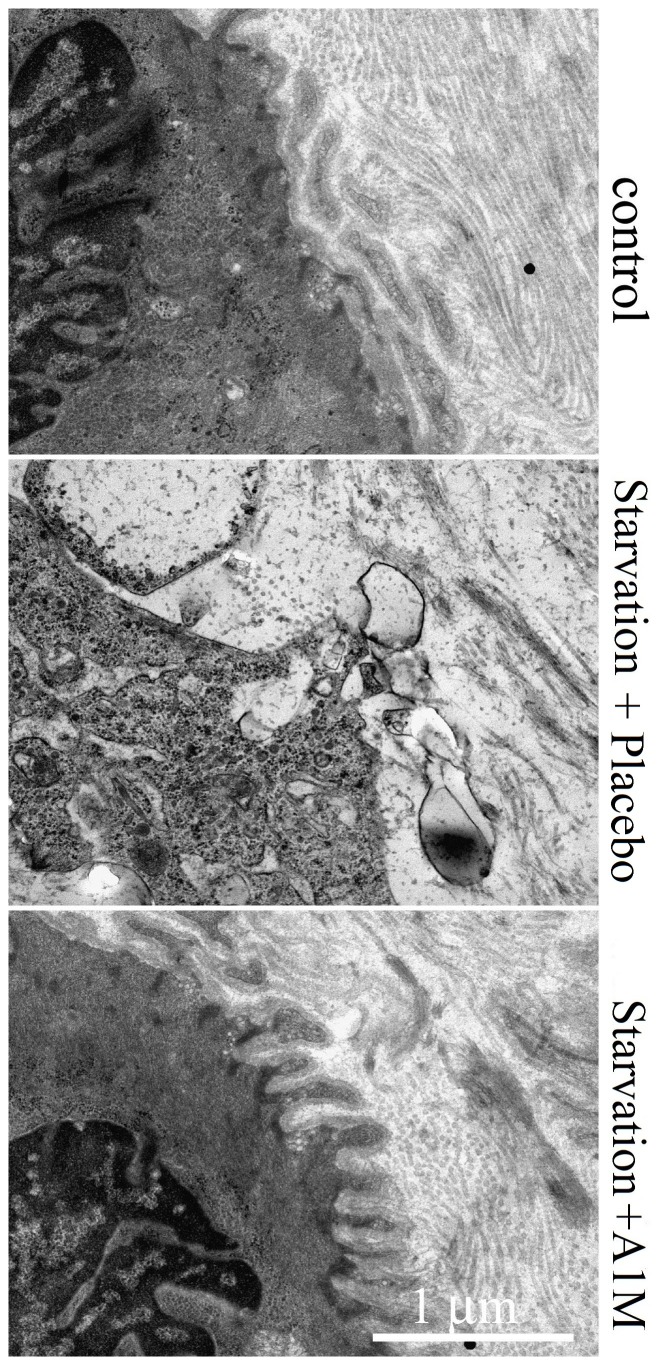
Placental ultra-stucture visualized by transmission electron microscopy. Ultra-structure of placental tissue visualized by transmission electron microscopy. The structure in ultra-thin sections of starved placebo (middle) and starved A1M treated (bottom) animals was compared to the control animals (top).

Electron microscopic analysis of the placental tissue showed that starvation caused severely compromised placental morphology. In particular there were signs of disrupted extracellular matrix (ECM) with considerable loss of extracellular collagen fibrils and other matrix proteins, leading to a denuded appearance of the extracellular space. Furthermore, starvation apparently induced a loss of placenta barrier integrity by compromising plasma and nuclear membrane integrity. Mitochondrial swelling, i.e. an increased mitochondrial cross section area was also observed, resembling an oxidative stress situation induced by free heme as previously described [11]. (Figure 6, Table 1). All these structural changes were significantly reversed by A1M treatment.

**Table 1:** Quantification of values for the surface areas obtained by transmission electron microscopy of the placenta tissue samples.

Structures	Control	Starved Placebo	Starved A1M
ECM integrity	91±6%	19±19%	75±9%
Plasma membrane integrity	95±4%	29±26%	81±8%
Nuclear membrane integrity	95±5%	33±23%	79±6%
Mitochondrial cross section area (µm^2^)	0.5±0.2	1.7±0.9	0.7±0.3

Data are presented as mean from 60 cell profiles ± S.D.

### Utero-placental blood flow

Doppler velocitometry showed that the median RI for the uterine artery was 0.69 (range 0.66–0.74) and 0.67 (range 0.60–0.84) in the A1M group and the placebo group, respectively (p<0.70). Notching was present in three cases in the placebo group but in no cases of the A1M group (p<0.46). No significant correlation was revealed between the uterine artery RI and maternal mean blood pressure or heart rate, respectively. In the umbilical artery, the median RI was 0.56 (range 0.53–0.60) in the A1M group and 0.54 (range 0.51–0.61) in the placebo group (p<0.52). There was no significant correlation between the umbilical artery RI and fetal heart rate in the two study groups.

### Kidney histology

**Figure 7:**
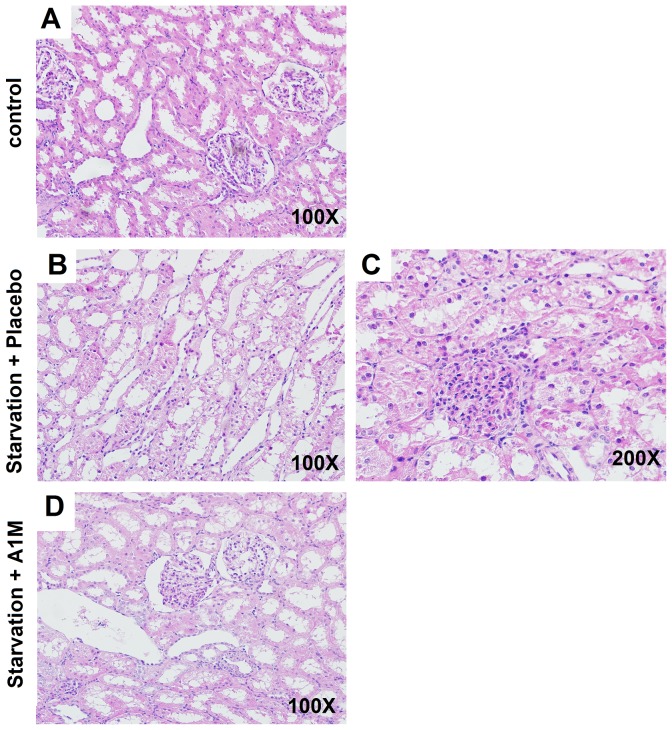
Renal histology in starved, placebo-treated ewes. The renal tissue was studied using light microscopy in cortical specimens stained with hematoxylin and eosin. A. Normal cortical tissue morphology is shown. The renal tubules show minor signs of postmortal changes but the height of the epithelium is normal, and only discrete cytoplasmal vacuolizations are seen. The glomeruli demonstrate open capillary loops and no signs of segmentation (magnification 100×). B and C. Distinct signs of acute tubular necrosis (ATN) are evident in the form of sloughing of apical plasma membranes and tubular cells into the lumen. In addition, a reduced epithelial height can be seen (B, 100× magnification). There are also signs of glomerular endothelial swelling seen from the closure of capillary loops and a non-isometric vacuolization of the tubular epithelium can be seen (C, 200× magnification). D. In the A1M-treated ewes, only small tubular changes compatible with ATN are present, but to a milder degree than in the placebo treated group. Also, no signs of glomerular endothelial swelling can be seen (100× magnification).

Histology sections of kidney cortex show signs of glomerular and tubular injury in starved animals (Figure 7). Signs of pronounced acute renal injury were present in the group subjected to starvation followed by placebo treatment. The tissue changes were typical for acute renal injury (ARI), with sloughing of apical plasma membranes, cytoplasmic vacuolization, and cellular drop-off from the basal lamina. Focally, glomeruli displayed signs of endothelial swelling resulting in segmentalization of the capillary tuft, which are indications of endothelial damage. In contrast, starved animals treated with A1M only showed slight signs of ARI, mostly in the form of apical sloughing of plasma membranes. Epithelial height was preserved and the cytoplasmic vacuolization was not as pronounced. No signs of glomerular segmentalization were seen.

**Figure 8:**
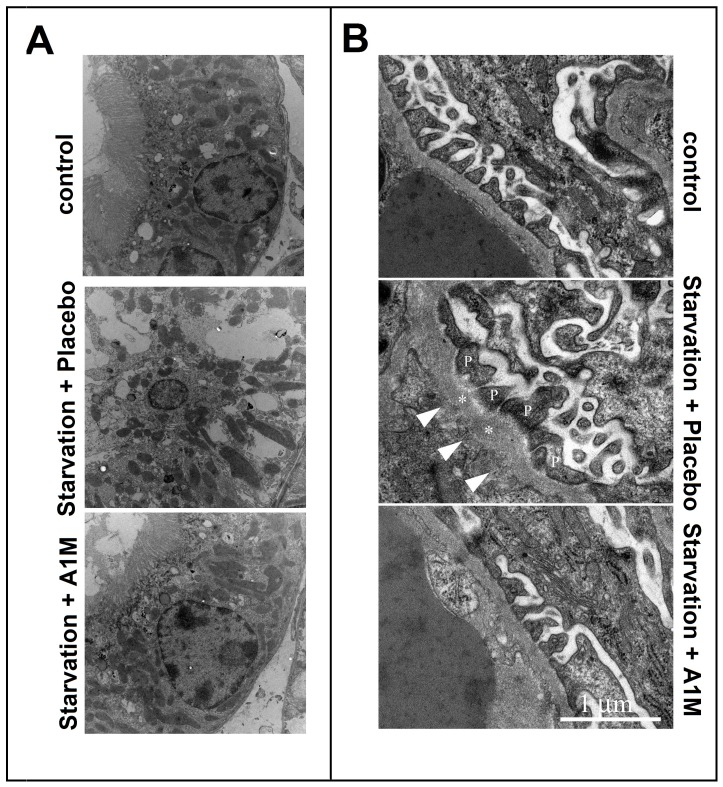
Ultra structure of renal tissue visualized by transmission electron microscopy. Panel A shows a representative areas of the proximal tubules with morphologically intact tubular cell linings seen in the control animals (upper panel) and in the starved animals treated with A1M (lower panel). In contrast, starvation and placebo treatment led to severe tubular damage and cell necrosis (middle panel). Panel B shows the glomerular area. The arrows point at abnormal regions on the basement membrane with fenestrations underneath. The podocytes show a disturbed morphology. The asterisks mark the basal membrane and P indicates the podocyte cell bodies.

EM analysis of kidneys from starved placebo- and A1M-treated animals, compared to the healthy control individuals showed the same result as electron microscopy of placenta tissue: starvation led to severe ultrastructural damage that was blunted by A1M treatment (Figure 8, Table 2) The podocytes exhibited a disturbed morphology, an irregular and contracted cell body leading to a ruffled appearance of the underlying basement membrane, after starvation. Podocyte necrosis was also frequently observed, leading to impaired glomerular barrier morphology with large basement membrane fenestrations (Figure 7B, arrowheads). Similar phenomena were observed in proximal tubules where starvation induced tubular damage with cell necrosis and an overall disturbed tubular morphology (Figure 7A, middle panel). These overall effects were reversed by A1M (Figure 7 A and B, lower panels).

**Table 2:** Quantification of values for the surface areas obtained by transmission electron microscopy of the kidney tissue samples.

Structures	Control	Starved Placebo	Starved A1M
ECM integrity	96±3%	41±21%	92±8%
Plasma membrane integrity	95±4%	36%±29	84±7%
Nuclear membrane integrity	96±3%	39%±23	89±9%
Mitochondrial cross section area (µm^2^)	0.4±0.2	1.7±1.1	0.6±0.3

Data are presented as mean from 60 cell profiles ±S.D.

### Kidney function

Starvation did not result in significant proteinuria (Table S5 in file S1).

**Figure 9:**
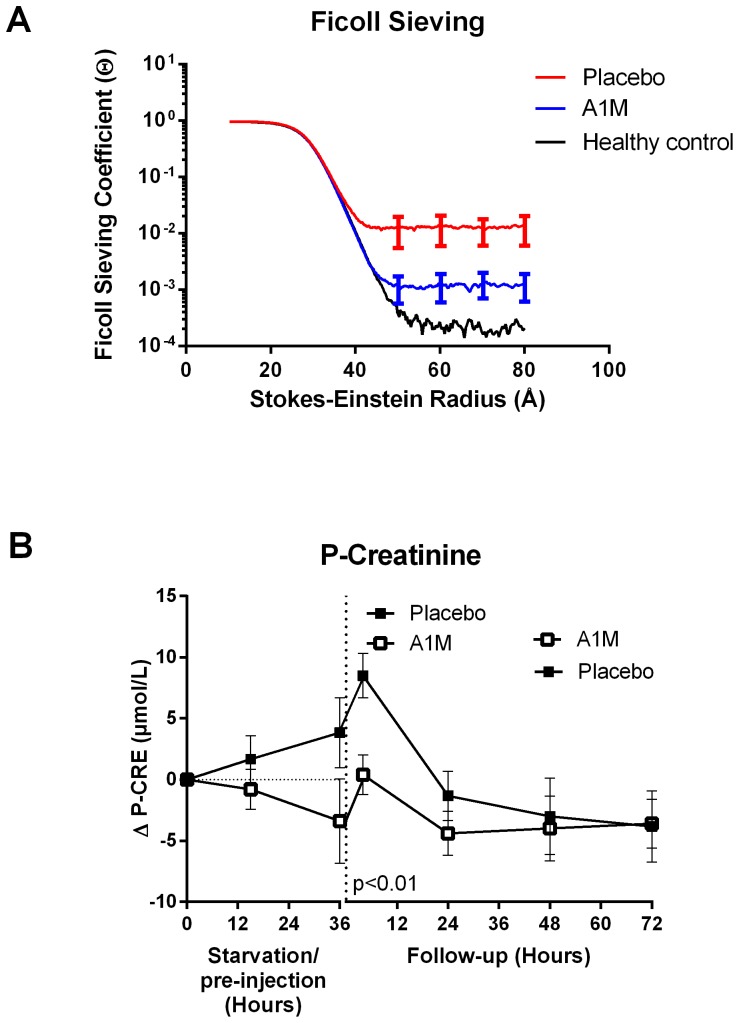
Kidney function. A. Glomerular sieving of FITC-Ficoll. The glomerular sieving coefficients (θ) plotted vs. Stokes-Einstein radius (a_e_), for Ficoll molecules ranging from 10 to 80Å in radius. A marked decrease in θ for large Ficoll molecules (Ficoll_50–80Å_) was seen for the starved group given A1M compared to the starved placebo group. For Ficoll_70 Å_, θ was 1.80×10^−3^±5.54×10^−4^ and 1.18×10^−2^±5.84×10^−3^ (p<0.055), starved placebo and starved+A1M respectively. The data are presented as the mean ±SEM and the p-value was calculated using un-paired t-test. B. Plasma creatinine levels. The plasma creatinine levels during the experiment, normalized to experiment start (0 h) -values. The arrows indicate the A1M injections. The data is presented as the mean ±SEM of Δ time point X-0h values. The p-values were calculated using un-paired t-test. The indicated p-value is valid for the 4 hour re-fed follow-up time point, which showed significant increase in the plasma creatinine level in the placebo treated animals.

The glomerular sieving coefficient for Ficoll_50–80Å_ was increased in the starved placebo treated animals (Figure 9A) indicating a defect in the glomerular filtration barrier following starvation. Treatment with A1M counteracted the increased sieving coefficients for Ficoll_50–80Å_. Plasma creatinine concentration increased significantly in the placebo treated animals at 4 hours of follow-up, but not in the A1M treated group (Figure 9B).

### Blood analysis

**Figure 10:**
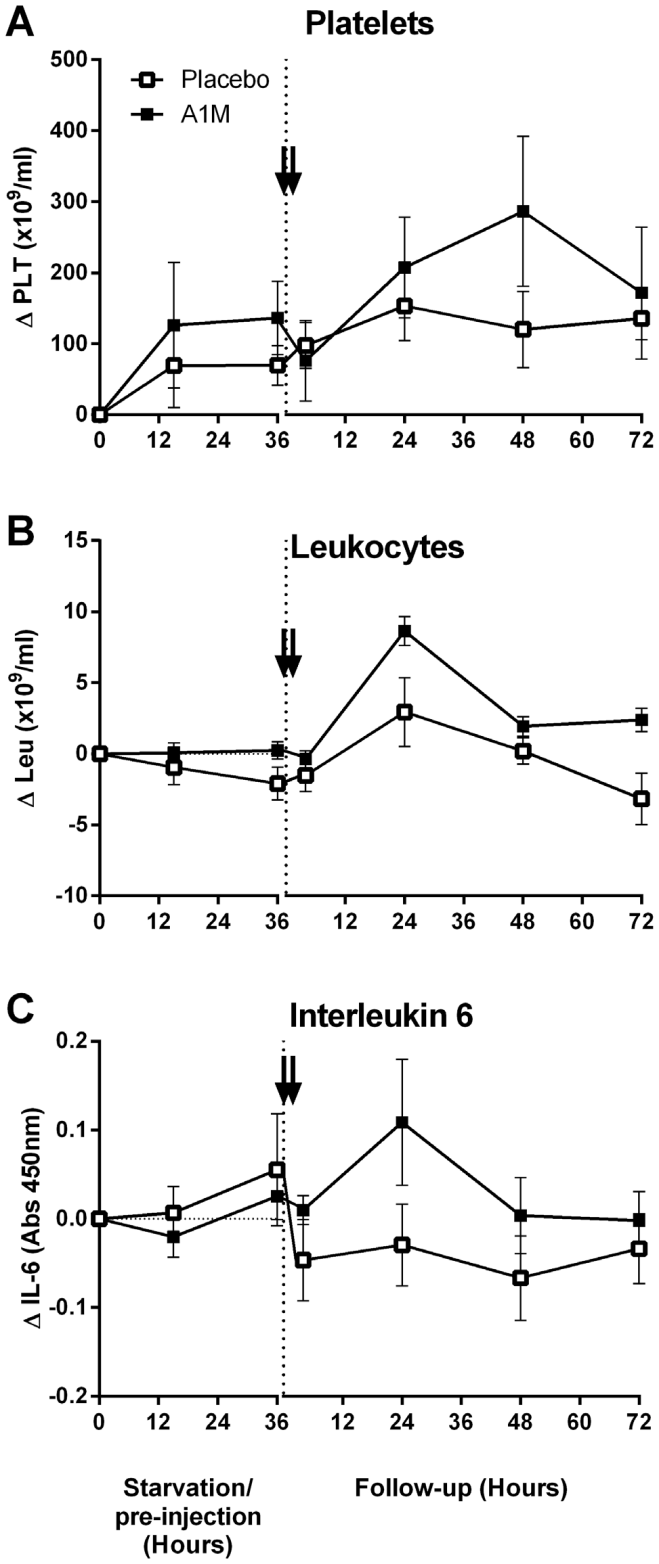
Blood cell distribution and interleukin 6 plasma concentration. A–B show the number of platelets and the total number of leukocytes in peripheral blood. Figure C shows the plasma levels of interleukin 6 as determined by ELISA. The arrows indicate the A1M injections. As there was a large individual variability already at the experimental start (0 h), the subsequent values for each time point for an individual were related to its own 0h-value. The data is presented as the mean ±SEM of Δ time point X-0h values. A1M treated animals vs their values at 0 h showed significantly increased platelets at 48 hours after the first injection (p<0.04) and of the total number of leukocytes at 24 hours after the first injection (p<0.0007). No significant changes were seen in the placebo-treated animals vs their 0 h values at any time-point. A small, non-significant, decrease of cells in the placebo-treated group led to a significantly higher number of total leukocyte in the A1M treated animals vs placebo treated at the end point (p<0.03). Statistical significance was calculated using unpaired t-test.

Starvation did not significantly change the number of blood cells (Figure 10 and Figure S2). However, A1M treatment resulted in a transient increase in the total number of platelets 48 hours after the first injection and peripheral white blood cells, monocytes and lymphocytes 24 hours after the first injection. The number of monocytes remained elevated until termination of the experiment. Treatment with A1M led to a small, non-significant, transient increase in plasma interleukin 6 24 hours after the first injection. Plasma TNFα did not show any significant alternations between the study groups.

### Gene expression analysis

**Figure 11:**
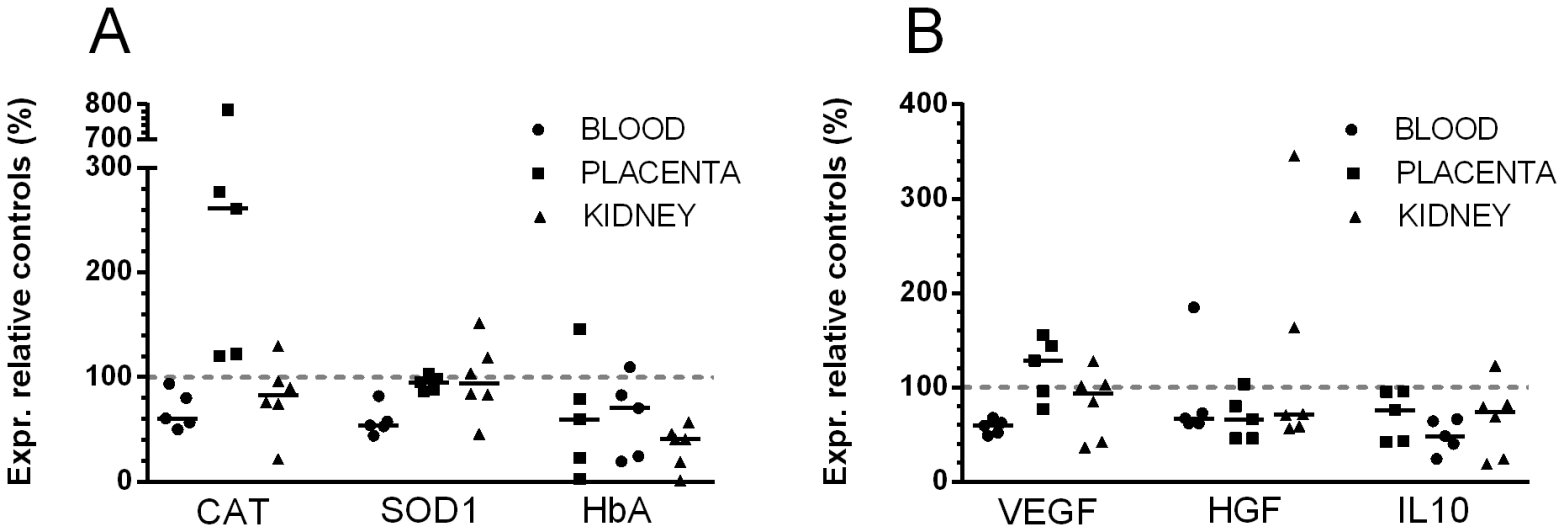
The effect of starvation on gene expression, in blood, placenta and kidney tissues. The target gene expression in pregnant healthy control ewes was analyzed and the median value was set to 100% (represented by a dash-dot line). The relative gene expression in the starved, placebo-treated ewes, are shown as scatter plots with medians. The levels are normalized to β-actin. A. The effect on oxidative stress genes and HbA. There were a significant decrease of CAT and SOD1 mRNA expression in white blood cells (p-values<0.043 and <0.008 respectively). The CAT mRNA expression was up-regulated 2.6-fold in the placenta and the HbA mRNA expression was down-regulated 3.3-fold in the kidneys. B. The effect on genes related to angiogenesis and inflammation. There was a significant decreases of VEGF, HGF and IL10 in white blood cells (p-values<0.02, <0.06 and <0.08, respectively). HGF and IL10 mRNAs expression showed down-regulation to comparable extent in the placenta, but not VEGF. In the kidneys, none of the genes were significantly affected by starvation.

**Figure 12:**
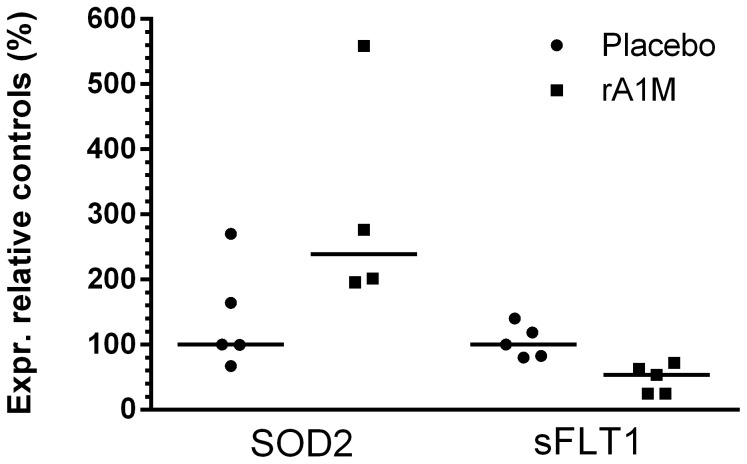
The effects of A1M-treatment on gene expression in white blood cells, placenta and kidney tissue. The A1M treatment regulates transcription of SOD2 and s-Flt1 genes in white blood cells in the starved pregnant ewes. The gene expression in starved ewes injected with placebo (•) or A1M (▪) were analyzed. The gene expression data was normalized to β-actin. The median value of the starved+placebo group was set to 100% and the results are expressed as percentage of the median value. The A1M treatment caused significant increase in SOD2 mRNA expression in white blood cells (p = <0.043) and a decreased expression of s-Flt1 mRNA (p = <0.012). All other investigated genes were unaffected by A1M treatment.

The differential expression of AMBP, HbA and HbF and a selection of genes related to oxidative stress, inflammation and angiogenesis were analysed in blood kidney and placental tissue of the starved ewes and two healthy pregnant control ewes. The effect of starvation as well as the effect of A1M was evaluated (Figure 11 and 12).

Starvation led to a significantly down-regulation of the oxidative stress-related SOD1 (p<0.008) and catalase (p<0.043) in blood, but a 2.6-fold up-regulation of catalase in placenta (Figure 10A). Also, starvation caused a down-regulation of HbA in kidneys. Furthermore, starvation led to a statistically significant or close to significant down-regulation of the inflammation and angiogenesis-related VEGF (p<0.02), HGF (p<0.06) and IL-10 (p<0.08) in blood cells (Figure 10B). In placenta the same trend was seen for HGF and IL10, but not for VEGF.

Treatment of starved ewes with A1M up-regulated the expression of SOD2 (p<0.043) and down-regulated the expression of s-Flt1 (p<0.012), in blood (Figure 11).

## Discussion

In this study, we have shown that starvation of pregnant ewes for 36 hours leads to a mild hemolysis. We also show damage on placenta and kidney tissue typical of oxidative stress and impaired kidney function as previously described for the model [26]. These toxic reactions were reversed by treatment with the heme and radical scavenger A1M.

The pregnant sheep model, in its original setting with 96 hours of starvation, displays pregnancy specific hypertension, proteinuria and endothelial dysfunction [24]. The disease is induced late in pregnancy, as a model of the endothelial dysfunction seen in stage 2 of PE. The mechanism is a starvation-induced lysis of the ewe erythrocytes that leads to release of free Hb to the circulation. Free Hb and its metabolites induces inflammation and oxidative stress [36], which damage the endothelium [37] and organs in general and kidneys in particular [38]. Despite the, somewhat artificial origin of free Hb, we believe that the induced stress-mechanisms operating in this model, are very similar to those in PE.

In the mild starvation model, 36 hours of starvation, used in this study a small increase of circulating heme and a two-fold increase of the more stable heme degradation product, bilirubin was measured. The amplitude of the bilirubin increase is in agreement with the original, 96 hours of starvation model [24], arguing for that a similar hemolysis was induced. Furthermore, the hemolysis led to oxidative stress, reflected by a decreased plasma concentration of free thiol groups and increased catalase expression in the placenta. The oxidative stress caused acute structural tissue damage in the placenta and the kidneys. The placenta showed severe structural damages such as disruption of extracellular matrix, plasma and nuclear membrane integrity and increased mitochondrial area. These finding are similar to those previously observed in *ex-vivo* Hb perfused placentas [11]. In the kidneys, vacuolization and signs of glomerular endotheliosis were seen. In addition to the structural damages to the kidneys, increased glomerular leakage was measured indicating impaired kidney function. The combined results from the study therefor show that a shorter starvation period,is sufficient to initiate hemolysis and a heme-induced oxidative stress that causes tissue and endothelial damages. However, the mild starvation was not enough to cause hypertension and proteinuria as described in the original model. Most likely, these symptoms need prolonged oxidative stress to develop and therefore only occur after a longer starvation period. In summary, despite the shorter starvation period, the current modified model was adequate for studies on free Hb induced oxidative stress reactions and the protective and therapeutic effects A1M.

Endogenous A1M is well conserved among species and normally found in blood and extra-cellularly in all tissues [39]. It is secreted from the liver into the blood and then rapidly distributed to the extravascular space of all organs [22]. Due to its small size, free A1M is also filtered by the glomeruli and then re-adsorbed in the tubuli. Thus, the half-life of A1M in the blood circulation is short [22]. In this study, injected A1M showed a quick turn-over, both in plasma and urine, as expected. In plasma, the concentration of exogenous A1M was approximately 10 µg/mL, two hours after injection. In rats, the endogenous concentration of A1M has previously been determined to 16 µg/mL [40]. Assuming a similar concentration in ewes, the injection of A1M therefore almost doubled the total A1M-concentration in plasma. However, only 10–15% of the original dose of A1M remained in plasma two hours post-injection and 0.25% and 1% was found in plasma and urine, respectively, after 24 hours. This suggests that most of the injected A1M was rapidly distributed to the extravascular space or excreted by the kidneys during the interval between 2 and 24 hours post-injection.

The injected A1M showed therapeutic effects in the placenta and the kidneys. In placenta, light microscopy showed a tendency of increased erythrophagocytosis in the starved, placebo treated animals, which was reversed by A1M-treatment. The general starvation-induced hemolysis caused accumulation of erythrocyte debris in the placenta that in turn increased phagocytosis, a mechanism previously described [41]. Treatment with A1M attenuated the amount of erythrocyte debris, possibly through its scavenging action. Further studies are needed to decipher the role of A1M in placental erythrocyte phagocytosis. In kidneys, the degree of vacuolization of the proximal tubules was milder after A1M-treatment compared to the starved placebo-treated group. In addition, the glomerular endotheliosis, observed in the starved placebo-treated animals, was absent in the A1M-treated group. In line with this, the starvation-induced impaired glomerular function (e.g. sieving coefficient and filtration rate) was significantly reversed by A1M-treatment.

The vascular effects of starvation and A1M-treatment were also evaluated by Doppler ultrasound. The Doppler analysis showed presence of notching in three of the starved cases receiving placebo but not in the A1M-treated group. Although not statistically significant, these findings may still suggest that starvation and increase in free Hb contributes to an increased vascular tone reflected by the notching, which appeared to be ameliorated by A1M treatment.

The therapeutic effects of A1M may act through several mechanistic pathways of which some will be discussed in detail below.

1. A1M is a reductase and radical scavenger that covalently traps organic radicals, heme being one of them [29], [42], [43], and thus protects cells and tissues from Hb-, heme- and ROS-induced cell-damage. The protective effects of A1M have previously been shown in several *in vitro* cell- and organ systems [11], [19], [23], [44], [45]. Antioxidation by reduction, heme binding and radical scavenging is therefore most likely the major protective mechanism in this investigation.
2. A1M was previously shown to bind to collagen fibres and mediate repair of Hb-, heme- and ROS-damaged collagen fibrillar structure [18], [44]. A similar ECM repair effect was seen in this study, both placenta and kidney showed normalized structure after treatment with A1M. Furthermore, in a previous report, human placentas perfused with free Hb displayed PE-like placental damage that were reversed by treatment with A1M, partially by up-regulation of extracellular matrix related genes possibly contributing to re-organization of the damaged extracellular matrix [11].
3. In addition to having anti-oxidative effects on its own, the current *in vivo* data suggests that A1M induces other antioxidant systems. The SOD2 gene expression was significantly up-regulated in white blood cells suggesting an important effect on immune cells *in vivo*.
4. The significant down regulation of the anti-angiogenic factor s-Flt1 seen in white blood cells was an unexpected effect of A1M. Imbalance between angiogenic and anti-angiogenic factors is well described in PE [46]. In fact, infusion of sFlt1 in rats is an established animal model for PE [47]. A new and important mechanism for A1M may therefore be to restore the angiogenic balance that is seen in PE by negatively affecting expression of s-Flt1. Due to lack of species-specific antibodies, the full spectrum of angiogenic and anti-angiogenic factors could not be analyzed in plasma.
5. A slight increase in the number of immune cells and blood platelets was observed in the A1M-infused sheep, peaking several days after injection. Considering that A1M was produced in an *E.coli* expression system, special care was taken to avoid bacterial contaminants such as endotoxin, protein or DNA. The content of endotoxins was carefully monitored and found to be <5 EU/mg. The ewes thus received less than 10 EU/kg body weight, twice within 2 hours, which corresponds to an endotoxin dose around the upper limit for an approved drug [48]. It is therefore unlikely that the increase in blood cell counts were induced by endotoxin contamination. A1M has previously been shown to have immune regulatory effects *in vitro*, mostly immunosuppressive, *i.e.* inhibition of immune cell stimulation [49]. Thus, it cannot be ruled out that immune regulation is mechanistically involved in execution of the therapeutic effects of A1M.

The important results from this first *in vivo* study are that (a) A1M is well tolerated by the pregnant ewes and yielded no clinical signs of adverse reactions or laboratory findings indicating toxic reactions to vital organs, and (b) A1M showed therapeutic effects in the pregnant ewe PE starvation model through several different possible mechanisms. The data in this work therefore support the use of A1M as a potential, efficient and safe drug for treatment of PE.

## Supporting Information

File S1 (DOCX)

Figure S1 (TIFF)

Figure S2 (TIFF)
