# Supplementary material for: A1M/α1-Microglobulin Protects from Heme-Induced Placental and Renal Damage in a Pregnant Sheep Model of Preeclampsia
Source: PLoS One. 2014 Jan 28;9(1):e86353. doi: 10.1371/journal.pone.0086353 (PMC3904882; doi:10.1371/journal.pone.0086353)
Supplement: File S1 — (DOCX) [file pone.0086353.s001.docx]

**Supplementary Table S1:**

Accession numbers, forward and reverse primer sequences, probe sequences, and exon boundaries for ovine transcripts analyzed with qPCR.

| Transcript | Accession number | Primers, forward and reverse | Probe | Exons |
| --- | --- | --- | --- | --- |
| ov. β-actin | NM_001009784.1 | Forward: GCTCTTCCAGCCGTCCTT  Reverse: TGAAGGTGGTCTCGTGAATGC | CCTGGGTATGGAGTCCTG | 2-3 |
| ov. CAT | GQ421282.1 | Forward: CATCTGAAAGACGCACAGCTT  Reverse: GATGCGGGAGCCATACTCA | AAGCGGTTAAGAACTTC | 12-13 |
| ov. SOD1 | NM_001145185.1 | Forward: GGGCCGGAGAGCCTTTC  Reverse: GGGCCGTCACCCTTCAG | TCGTCGCCATGACTCG | 1-2 |
| ov. SOD2 | GQ221055.1 | Forward: CGTCGCCGAGGAGAAGTAC  Reverse: GCAGGCTGCAGAGCTATCT | CTGTGACATCACCCTTCTC | 2-3 |
| ov. HbF  (γ-chain) | UO1378.1 | Forward: GGCTTCTGTCATCTCCCTATTTGC  Reverse: GTGCTCGAAGAACCTCTGAGT | CAACCAGCAGCACCTTG | 1-2 |
| ov. HbA  (α-chain | EU938073.1 | Forward: TGCACGCCCACAAGCT  Reverse: AGGGTCACCAGCAGGGT | TCAGAAGCTTGAAGTTGACC | 1-2 |
| ov. PLGF | AY157708.1 | Forward: CCCTGGAGACAGCCAACG  Reverse: GGCTGGTCCAGAGAGTGGTACT | CCATGCAGCTCATG |  |
| ov. s-Flt1 | AF488351 | Forward: GACTGTGGAAAGAAACGTGAACTT  Reverse: CATTTTGTCCAAGCTCACCTGAA | CACAGATGTGCCCCATGGAT | 12-13 |
| ov. VEGF | X89506 | Forward: GGATGTCTACCAGCGCAGC  Reverse: TCTGGGTACTCCTGGAAGATGTC | TCTGCCGTCCCATTGAGACCCTG |  |
| ov. TGFβ | NM_001009400 | Forward: AAGCGGAAGGGCATCGA  Reverse: CGAGCCGAAGTTTGGACAAA | CCATCCGCGGTCAGA |  |
| ov. HGF | AF213397.1 | Forward: TCAAATGCCAGCCCTGGAAT  Reverse: GTCTTTACCCCGATAGCTCGAA | CACATGAACACAGCTTTT | 4-5 |
| ov. IL-6 | NM_001009392 | Forward: AGGAAAAAGATGGATGCTTCCA  Reverse: GACCAGCAGTGGTTTTGATCAA | CTGGGTTCAATCAGGCGA |  |
| ov. IL-10 | NM_001009327.1 | Forward: CATGCTGTTGACCCAGTCTCT  Reverse: GGTAAAACTGGATCATTTCCGACAAG | CCAGGTAACCCTTAAAGTC | 2-3 |
| bov HMOX1 | NM_001014912.1 | Bt03218621_m1 |  |  |
| bov Ambp | NM_173989.2 | Bt03212235_m1 |  |  |

**Supplementary Table S2:** Ewe parameters.

| **Parameter** | **Placebo group** | **A1M group** |
| --- | --- | --- |
| Ewe age (years) | 3 (2.8-8)^a^ | 3 (2.5-5.5)^a^ |
| Ewe weight (kg) | 75 (68-82)^b^ | 76 (69-84)^b^ |
| Gestation day | 130 (129-130)^a^ | 130 (128-131)^a^ |

^a^ Median (25% percentile-75% percentile)

^b^ Mean (95% confidence interval)

**Supplementary Table S3:** Lamb parameters.

| **Parameter** | **Placebo group** | **A1M group** |
| --- | --- | --- |
| Number of off-spring per Ewe | 1.5 (1-2.25)^a^ | 3 (1.5-3)^a^ |
| Total weight of off-spring per Ewe (g) | 4700 (2400-7000)^b^ | 7300 (4200-10400)^b^ |
| Weight per off-spring (g) | 2800 (2200-3400)^b^ | 3000 (2400-3500)^b^ |

^a^ Median (25% percentile-75% percentile)

^b^ Mean (95% confidence interval)

**Supplementary Table S4:** Starvation parameters.

| **Parameter** | **0h** | **15h** | **36h** |
| --- | --- | --- | --- |
| Blood glucose (mmol/L) | 2.3 (1.5-3)^a^ | 1.8 (1.3-2.2) | 1.6 (1-2.1) |
| Urine-pH | 8.5 (7.9-9.2) | 8.1 (7.3-8.9) | 6.6 (5.8-7.4)  *(p<0.004)^b^* |
| Plasma-Ca^2+^ (mmol/L) | 2.16 (2.04-2.28) | 2.13 (1.98-2.28) | 2.05 (1.93-2.17) |
| Urine-ketones (mg/dL) | 55 (21-90) | 62 (29-95) | 102 (66-136) *(p<0.11)* |
| Plasma- heme (µM) | 14.2 (12.7-18.1) | 14.9 (12.9-16.9) | 15.2 (13.4-17.1) |
| Plasma-bilirubin (µM) | 3.2 (2.2-4.3) | 5.1 (3.3-6.8)  *(p<0.06)* | 6.5 (5.0-8.0)  *(p<0.001)* |
| ΔPlasma-free thiols^c^ (ΔA_405nm_) | 0 | -11.84 (-18.8- -4.9) | -11.82 (-24.4- 0.75)  *(p<0.04)* |

^a^Mean (95% confidence interval).

^b^p-values for the change in comparison to the 0h time point were calculated by unpaired t-test and are presented in parentheses in italic.

^c^The free thiols are presented in relation to the 0h-value.

**Supplementary Table S5:** Incidence of positive detection in urine.

Positive detection of urine protein, leukocytes and erythrocytes, respectively are shown. Positive detection is defined as: for protein (>25mg/dL), leukocytes (>15cells/µl), erythrocytes (>10cells/µl).
