# Supplementary figures and images for: A1M/α1-Microglobulin Protects from Heme-Induced Placental and Renal Damage in a Pregnant Sheep Model of Preeclampsia
Source: PLoS One. 2014 Jan 28;9(1):e86353. doi: 10.1371/journal.pone.0086353 (PMC3904882; doi:10.1371/journal.pone.0086353)

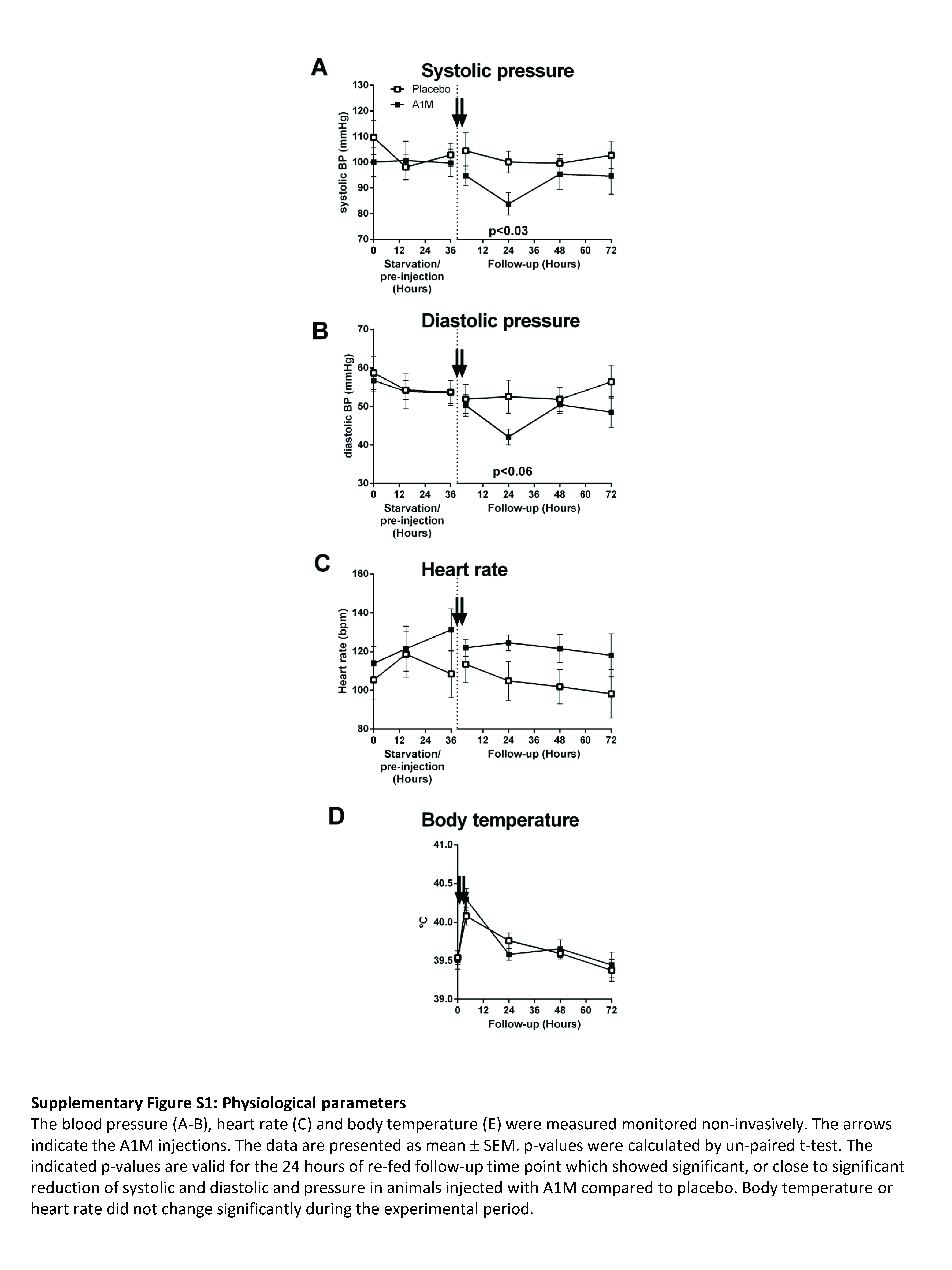

Supplement: Figure S1 — (TIFF) [file pone.0086353.s002.tiff]

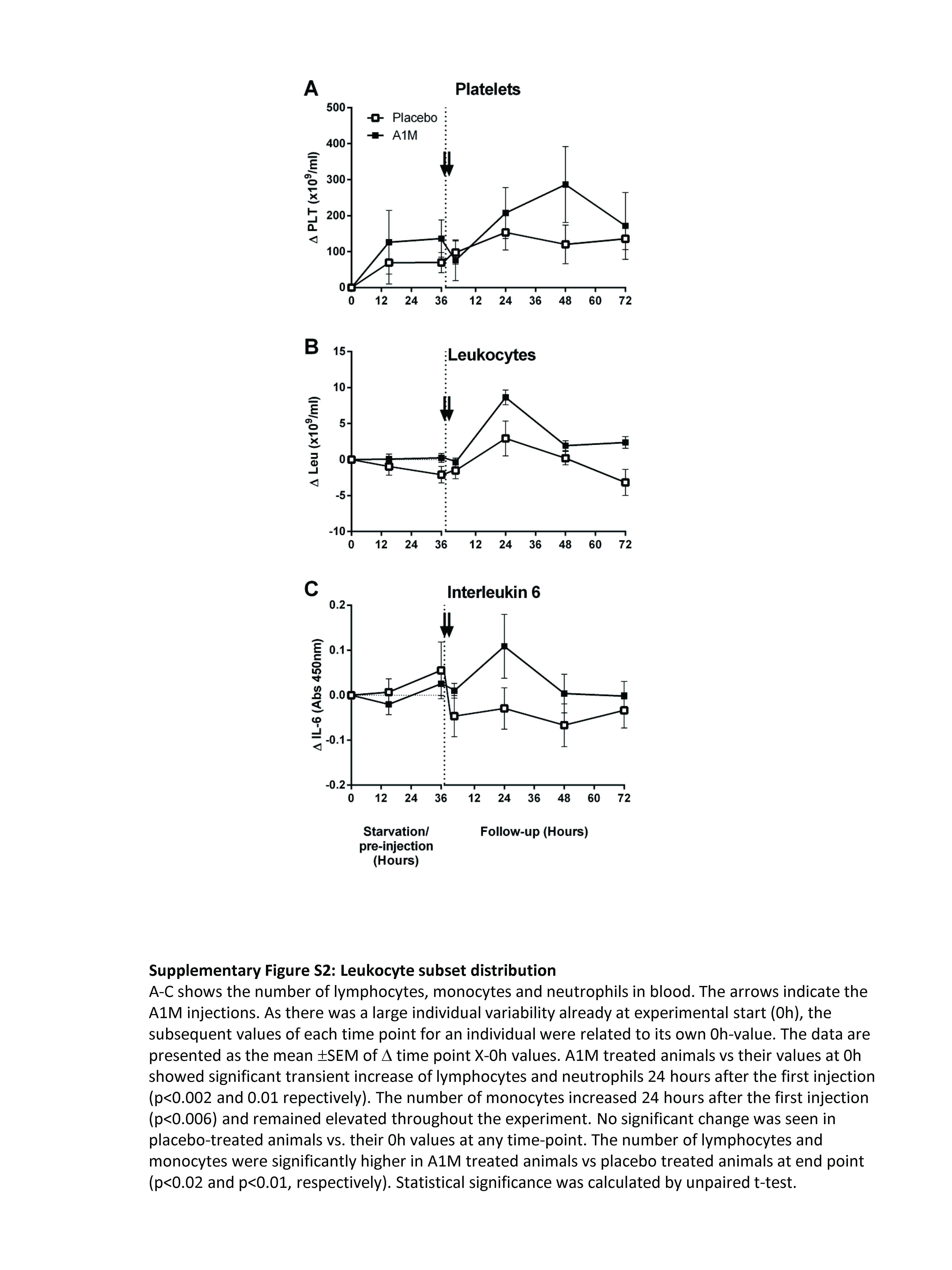

Supplement: Figure S2 — (TIFF) [file pone.0086353.s003.tiff]
